# Microalbuminuria as a Predictor of Early Neurological Deterioration and Poor Functional Outcomes in Acute Ischemic Stroke

**DOI:** 10.7759/cureus.58311

**Published:** 2024-04-15

**Authors:** Krishna Sai Pavuluri, Debasis Pathi, Santosh Kumar Dash, Pragateshnu Das, Sudhansu S Panda

**Affiliations:** 1 General Medicine, Kalinga Institute of Medical Sciences, Bhubaneswar, IND; 2 Neurology, Kalinga Institute of Medical Sciences, Bhubaneswar, IND; 3 Internal Medicine, Apollo Hospitals, Bhubaneswar, IND

**Keywords:** microalbuminuria, early neurological deterioration (end), acute ischemic stroke (ais), modified rankin scale (mrs), national institutes of health stroke scale (nihss)

## Abstract

Background

Ischemic stroke is a major health crisis with significant consequences. Microalbuminuria, a sign of endothelial dysfunction, has been linked to adverse outcomes in ischemic stroke. Early neurological deterioration (END) is a critical factor influencing the patient’s prognosis. This study aimed to determine the prevalence of microalbuminuria, its predictive value in assessing END, and its prognostic implications in acute ischemic stroke (AIS).

Methodology

This study conducted at Pradyumna Bal Memorial Hospital, Kalinga Institute of Medical Sciences Bhubaneswar (November 2020-April 2022) included 114 AIS patients over 18 years who presented within 24 hours of stroke onset. Demographics, vascular risk factors, National Institutes of Health Stroke Scale (NIHSS) scores (admission and day three), modified Rankin scores (day 10), urinary albumin-to-creatinine ratios, and carotid artery Doppler studies were collected.

Results

The mean age of the patients was 61.87 years, with males constituting 72.8% of the population. Hypertension (50.9%) and diabetes mellitus (28.9%) were the most common comorbid conditions. The mean NIHSS stroke severity at presentation was 11.30. END occurred in 38.6% of patients. Overall, 43.9% of cases showed carotid stenosis, and the mean carotid intimal media thickness was 1.08 mm. Notably, the presence of microalbuminuria significantly increased the chances of both END (39.45 times higher risk) and worse functional outcomes (odds ratio = 19.147, p = 0.001).

Conclusions

Microalbuminuria emerges as a robust independent predictor of END and a poor prognosis in AIS. These findings highlight the importance of early microalbuminuria identification and intervention to reduce END risk and potentially improve outcomes in AIS patients.

## Introduction

Ischemic stroke, a devastating global health crisis, is the leading cause of disability and a major contributor to mortality [1]. Microalbuminuria, the presence of small amounts of albumin in urine, indicates early endothelial dysfunction and is a marker of vascular damage [2]. Studies report a higher prevalence of microalbuminuria in ischemic stroke patients (14%-60%) compared to healthy individuals [3]. Early neurological deterioration (END), a significant worsening of neurological function within hours or days of a stroke, severely impacts patient outcomes [4,5]. Predictors of END include large infarct size, severe intracranial artery stenosis, specific lesion locations, high low-density lipoprotein cholesterol, and diabetes mellitus [6,7]. Emerging evidence suggests a potential link between microalbuminuria and both END and poor post-stroke outcomes. This prospective observational study aims to explore the predictive value of urinary microalbuminuria in assessing END and the short-term prognosis following an ischemic stroke.

## Materials and methods

This prospective observational study was conducted at a single center to investigate the role of microalbuminuria as a predictor and prognostic marker in individuals who had experienced an acute ischemic stroke (AIS). The study was conducted in the inpatient setting of the Department of Internal Medicine at Pradyumna Bal Memorial Hospital, Kalinga Institute of Medical Sciences (KIMS), Bhubaneswar, after obtaining ethical clearance (approval number: IEC/498/2020) and written informed consent from the patients.

The study population consisted of 114 consecutive AIS patients admitted between November 2020 and April 2022 who met specific inclusion and exclusion criteria. Previous research findings on the prevalence of END in stroke served as the basis for calculating the sample size. The inclusion criteria included patients aged over 18 years and admitted within 24 hours of stroke onset. All patients underwent a CT of the brain to confirm the ischemic stroke. MRI of the brain was done in negative CT cases. Patients were excluded if they had any history of ischemic or hemorrhagic stroke, transient ischemic attack, conditions known to influence urinary protein excretion (e.g., congestive heart failure, obstructive uropathy, urinary tract infection), chronic kidney disease, decompensated liver cirrhosis, febrile conditions, or were currently on angiotensin-converting enzyme inhibitor/angiotensin receptor blocker therapy.

We initiated a comprehensive evaluation process after obtaining informed consent. This included detailed history taking, physical and systemic examinations, and collecting relevant data such as demographics, vascular risk factors, kidney function status, hemoglobin A1c, white blood cell count, blood pressure, and thrombolytic therapy status. Neurological severity was assessed on the day of admission and again on day three using the National Institutes of Health Stroke Scale (NIHSS) to monitor END. This scale evaluates various neurological functions ranging from 0-42, with a change of 2 or more points from the day of admission signifying END. The urinary albumin-to-creatinine ratio was used to measure urinary microalbuminuria. Functional outcomes were assessed on day 10 using the modified Rankin scale (MRS), where scores of 4-6 indicated a poor outcome and 0-3 a good outcome. Additionally, all participants underwent a carotid Doppler study to assess carotid intima-media thickness (CIMT) as an atherosclerosis indicator and to evaluate carotid artery stenosis.

Statistical analysis was done using Stata version 15.1 software (StataCorp., College Station, TX, USA). The Wilcoxon rank-sum test was used to compare scores between groups, while the chi-square test was used to analyze categorical parameters. Multivariate regression analysis was used to identify independent predictors of END and early functional outcomes. A significance level of 1% was maintained throughout the analysis.

## Results

This comprehensive 18-month, single-center, prospective, observational, comparative study, conducted at the Department of General Medicine, Pradyumna Bal Memorial Hospital, KIMS Bhubaneswar, meticulously enrolled 114 subjects with AIS. Demographic analysis revealed a mean age of 61.87 ± 14.38 years, ranging from 19 to 94 years. Notably, the majority of participants were clustered in older age groups: 38.6% were between 46 and 60 years old, 32.5% were between 61 and 75 years old, and 21.1% were over 75 years old. The majority were males, constituting 72.8% (83) of the cohort, and females constituting 27.2% (31).

Following type 2 diabetes mellitus at 28.9% (33), hypertension was the comorbidity with the highest prevalence rate at 50.9% (58). Smoking and alcohol consumption were documented in 36.8% (42) and 20.2% (23) of patients, respectively. The mean systolic and diastolic blood pressure readings were 142.16 ± 26.88 mmHg and 89.93 ± 14.30 mmHg, respectively. Microalbuminuria was present in a significant 61.4% (70) of the cohort, with a mean of 97.72 ± 49.1 mg/g of creatinine. We observed a significant correlation between older age and microalbuminuria (p = 0.000), indicating a higher incidence of microalbuminuria in elderly patients compared to younger ones.

**Figure 1:**
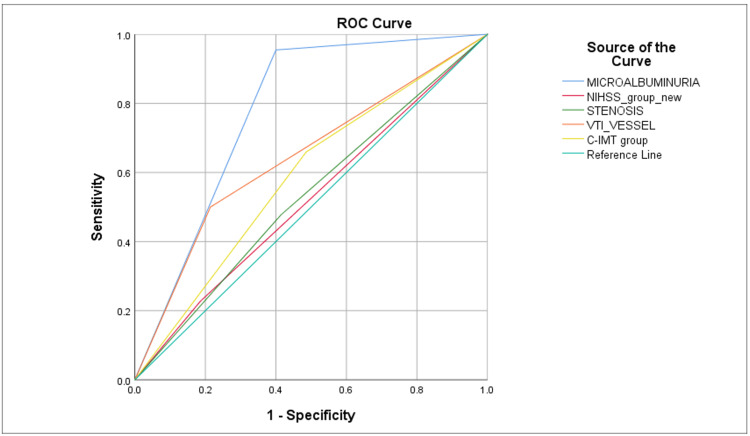
ROC showing AUC for variables associated with early neurological deterioration. ROC = receiver operating characteristic; AUC = area under the curve; NIHSS = National Institutes of Health Stroke Scale; CIMT = carotid intima-media thickness

**Figure 2:**
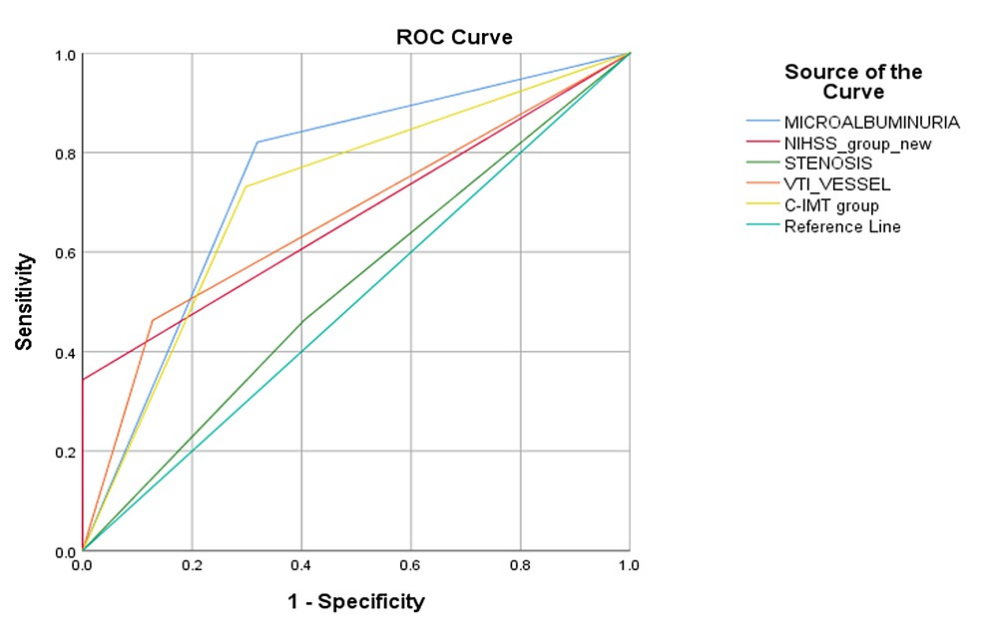
ROC showing AUC for variables associated with poor early functional outcome (MRS = 4-6). ROC = receiver operating characteristic; AUC = area under the curve; MRS = modified Rankin scale; NIHSS = National Institutes of Health Stroke Scale; CIMT = carotid intima-media thickness

Stroke severity was meticulously evaluated using NIHSS. On admission, the mean initial NIHSS score was 11.30 ± 6.24, ranging from 1 to 27. Based on day one NIHSS, 79.8% (91) of patients had a mild-to-moderate stroke (NIHSS < 15), while 20.2% (23) fell into the severe stroke category (NIHSS > 15). By day three, this score had increased to an average of 12.26 ± 8.08, ranging from 0 to 30, suggestive of END, as observed in 38.6% (44). In patients with END, microalbuminuria was present in 95% of the cases (Table 1). In contrast, 61.4% (70) of patients did not exhibit END. Multivariate regression analysis to predict END showed that severe stroke (NIHSS score ≥ 15) was an independent risk factor negatively correlated with END (p = 0.038), i.e., patients who had END had a lower NIHSS score on presentation. In our study, the factors that were significantly linked to microalbuminuria were older age (p = 0.000), high blood pressure (p = 0.004), severe stroke (NIHSS score ≥ 15) (p = 0.005), unfavorable (MRS 4-6) (p = <0.001), END (p = 0.001), thickened CIMT (≥1 mm) (p = 0.001), and large-vessel disease (p = 0.005). Further multivariate regression analysis revealed that microalbuminuria was independently associated with END (p = 0.00) and had a 39.45 times higher risk of developing END (Table 2). Upon doing a receiver operating characteristic (ROC) analysis among independent risk factors for END, microalbuminuria had the highest area under the curve (AUC) (p = 0.000) (Figure 1). Stroke outcomes were further evaluated on day 10 using the MRS, revealing a divergence between favorable outcomes (MRS = 0-3) in 41.2% of patients and unfavorable outcomes (MRS = 4-6) in 58.8%. Notably, microalbuminuria was present in 82% of those with an unfavorable outcome. In multivariate logistic regression analysis, microalbuminuria was independently associated with an unfavorable early outcome in patients with AIS (p = 0.001) (Table 3). Patients with microalbuminuria were at 19.147 times higher risk of developing an early, unfavorable outcome. On performing ROC analysis among independent risk factors for an early unfavorable outcome, microalbuminuria had the highest AUC (p = 0.000) (Figure 2).

**Table 1:** Analysis of the variables associated with microalbuminuria in the study population. *: variables showing significant association with microalbuminuria. HTN = hypertension; T2DM = type 2 diabetes mellitus; MRS = modified Rankin scale; END = early neurological deterioration; CA = carotid artery; CIMT = carotid intima-media thickness; NIHSS = National Institutes of Health Stroke Scale

Baseline variables	Categories	Microalbuminuria		Total	P-value
		No (n = 44)	Yes (n = 70)		
Age (median) *		52 years	69 years		0.000
Gender	Male	29	54	83	0.189
	Female	15	16	31	
HTN*	Yes*	15	43	58	0.004
T2DM	Yes	9	24	33	0.113
Smoking	Yes	13	29	42	0.200
Alcohol	Yes	7	16	23	0.368
MRS outcome*	Favorable (MRS = 0–3)	32	15	47	<0.001
	Unfavorable (MRS = 4–6)*	12	55	67	
END*	Yes	2	42	44	<0.001
Stenosis of CA*	Yes	11	39	50	0.001
CIMT*	Not thickened (<1 mm)	28	23	51	0.001
	Thickened (≥1 mm)*	16	47	63	
NIHSS severity*	Not severe <15	41	50	91	0.005
	Severe ≥15*	3	20	23	
Cerebral vessel size*	Small	37	40	77	0.005
	Large*	7	30	37	

**Table 2:** Multivariate analysis of variables to predict END. *: significant correlation. HTN = hypertension; T2DM = type 2 diabetes mellitus; END = early neurological deterioration; CIMT = carotid intima-media thickness; NIHSS = National Institutes of Health Stroke Scale; SE = standard error; OR = odds ratio; CI = confidence interval

Variables	Logit coefficient	SE	P-value	OR (95% CI)
Age	0.012	0.025	0.619	1.012 (0.965–1.062)
Female gender	-0.83	0.617	0.179	0.436 (0.13–1.461)
HTN	-0.776	0.637	0.223	0.46 (0.132–1.604)
T2DM	0.865	0.614	0.159	2.375 (0.713–7.914)
Severe NIHSS ≥15*	-1.404	0.676	0.038	0.246 (0.065–0.925)
Carotid stenosis	-0.785	0.585	0.179	0.456 (0.145–1.435)
CIMT (≥1 mm)	0.371	0.635	0.559	1.45 (0.418–5.029)
Large-vessel disease	0.832	0.597	0.163	2.299 (0.714–7.402)
Microalbuminuria*	3.675	0.942	0.0	39.45 (6.221–250.2)

**Table 3:** Multivariate regression analysis of variables to predict early poor outcomes. *: significant correlation. HTN = hypertension; T2DM = type 2 diabetes mellitus; CIMT = carotid intima-media thickness; SE = standard error; OR = odds ratio; CI = confidence interval

Variables	Logit coefficient	SE	P-value	OR (95% CI)
Age	-0.001	0.03	0.982	0.999 (0.943–1.059)
Female gender*	2.253	0.76	0.003	9.52 (2.148–42.197)
HTN	-1.354	0.729	0.063	0.258 (0.062–1.078)
T2DM	1.193	0.707	0.092	3.297 (0.824–13.188)
Carotid stenosis	-0.715	0.638	0.263	0.489 (0.14–1.709)
CIMT (thickness ≥ 1 mm)*	2.605	0.719	0.0	13.534 (3.307–55.383)
Large-vessel disease*	2.564	0.776	0.001	12.986 (2.839–59.401)
Microalbuminuria*	2.952	0.849	0.001	19.147 (3.625–101.144)

The carotid artery Doppler result showed that the mean CIMT was 1.08 ± 0.41 mm, ranging from 0.46 to 3.80 mm. The right carotid artery showed a slightly higher mean CIMT of 1.00 ± 0.41 mm compared to the left 0.97 ± 0.27 mm. Overall, 43.9% of the study population had carotid artery stenosis, with the right side affected in 21.1%, the left in 13.2%, and both in 9.6% of cases.

The ischemic strokes primarily affected the left side (52.6%), followed by the right side (43.9%), and only a small percentage (3.5%) affected both sides. The middle cerebral artery territory (69.3%), the anterior cerebral artery territory (3.5%), and the posterior circulation (16.7%) were the areas most frequently affected. Overall, 67.55% of patients had small-vessel disease, while 32.45% experienced large-vessel disease in our patients.

## Discussion

Increased urinary microalbumin is recognized as a significant risk factor for cerebrovascular diseases. Prior studies have shown that proteinuria elevates the risk of stroke severalfold compared to individuals without proteinuria, suggesting the potential of microalbuminuria as a biomarker for stroke susceptibility [8,9]. Our study sought to investigate the prevalence of microalbuminuria in ischemic stroke, its ability to predict END, and its impact on early functional outcomes following AIS.

Our findings revealed a 61.4% prevalence of microalbuminuria within our cohort, consistent with previous studies [10,11]. Importantly, microalbuminuria demonstrated a significant predictive value for END, with 95% of patients experiencing END also exhibiting microalbuminuria. These findings align with prior studies [12-14]. Multivariate regression analysis further solidified microalbuminuria as a robust, independent risk factor for END, revealing that individuals with microalbuminuria were approximately 39.45 times more likely to experience END. This underscores the findings of previous research [15-17].

Moreover, ROC analysis identified microalbuminuria and large-vessel disease as key predictors of END, highlighting the superior diagnostic accuracy of microalbuminuria. Our findings reinforce and supplement previous studies that propose a connection between artery size and various END risks but not a direct relationship between vessel size and END [18]. These findings underscore the importance of microalbuminuria as an independent predictor of END risk among AIS patients, suggesting it can play a vital role in clinical evaluations and strategies aimed at preventing END and ensuring favorable early outcomes.

We observed an inverse correlation between NIHSS scores and the incidence of END, implying that patients with milder initial strokes may be more prone to END, a finding consistent with some previous studies [19]. However, contrasting evidence suggests higher END rates in patients with more severe baseline NIHSS scores [20]. More research is needed to elucidate this relationship.

Our study used the MRS for outcome categorization to investigate the link between microalbuminuria and early functional outcome post-AIS. Favorable outcomes (MRS = 0-3) were achieved by 41.2% of our cohort, while 58.8% experienced unfavorable outcomes (MRS = 4-6). Multivariate regression analysis highlighted several independent predictors of poor outcomes, including female gender, carotid intimal thickness ≥1 mm, large-vessel disease, and, crucially, microalbuminuria. The presence of microalbuminuria was independently associated with an approximately 19-fold increase in the risk of a poorer outcome, echoing the results of previous studies [21-23]. The ROC analysis demonstrated that microalbuminuria outperformed other risk factors in predicting poor early functional outcomes, as evidenced by its higher AUC. These results align with studies highlighting microalbuminuria as an independent prognostic marker for adverse outcomes and increased short-term mortality [24,25].

Our study observed a mean CIMT of 1.08 mm, ranging from 0.46 mm to 3.80 mm. Carotid artery stenosis was present in 43.9% of cases, primarily affecting the right side. A CIMT ≥1 mm was significantly associated with the presence of microalbuminuria and independently predicted an unfavorable early functional outcome in ischemic stroke patients, increasing the risk by 19.14 fold. These findings support prior research demonstrating a strong correlation between increased CIMT and the risk of adverse stroke outcomes [26,27].

In our study, the majority of ischemic strokes (52.6%) occurred on the left side of the brain, primarily affecting the middle cerebral artery region (69.3%), leading to aphasia and significant functional impairment in our patients. Echoing previous studies, our results also showed that vessel size is not directly associated with END but increases the risk of a poor outcome by 12.9 times [28-30].

Our study has some limitations. Its single-center design may restrict the generalizability of the results, and as an observational study, it cannot establish causality. The exclusion criteria may have excluded patients with differing risk profiles for microalbuminuria. A larger study with a broader design and multiple measurement points is warranted.

## Conclusions

This study confirms the significance of microalbuminuria as an independent predictor of END and unfavorable outcomes in AIS patients. It highlights a marked increase in the risk of poor functional early outcomes and more chances of early deterioration in patients with microalbuminuria than without microalbuminuria, underscoring its role as a systemic marker of vascular health, especially in cerebral arteries.
